# Intra‐annual energy density cycles of spring‐ and fall‐spawning Atlantic herring *Clupea harengus* reveal different reproductive allocation tactics

**DOI:** 10.1111/jfb.70400

**Published:** 2026-03-17

**Authors:** Joseph B. Warren, Mark J. Wuenschel, Kenneth Oliveira

**Affiliations:** ^1^ University of Massachusetts Dartmouth North Dartmouth Massachusetts USA; ^2^ IBSS Corporation for NOAA Fisheries, Northeast Fisheries Science Center Woods Hole Massachusetts USA; ^3^ NOAA Fisheries, Northeast Fisheries Science Center Woods Hole Massachusetts USA

**Keywords:** Atlantic herring, bioenergetics, energetic condition, forage fish, reproduction, spawning seasonality

## Abstract

Atlantic herring *Clupea harengus* are total spawners that exhibit a large degree of reproductive plasticity and have substantial intra‐annual variation in their energetic condition. Recent research suggests that the species may be declining in energetic condition in the northwest Atlantic Ocean from the few historical records, but comparisons in the region are complicated by the presence of spring‐ and fall‐spawning contingents. Proximate composition analysis (water, lipid, protein and ash) results of Atlantic herring somatic tissue and gonads revealed sex‐specific energy allocation patterns and generated strong predictive relationships used to estimate the energy density (ED; kJ g^−1^ wet weight) of 1104 individuals collected between 2021 and 2023 by fishery‐dependent and ‐independent sources. Generalized additive models showed non‐linear somatic ED cycles that varied by spawning contingent and day of year. The best model included an interaction between day of year and spawning contingent, and explained 64.6% of the deviance, 12.2% more than the model without spawning contingent. Gulf of Maine and Georges Bank Atlantic herring ED was less variable in the spring compared to the fall, when there was a smaller energetic offset between contingents. Estimates of contingent‐specific seasonal somatic ED cycles provide context for interpreting values across studies, evaluating long‐term energetic condition trends and assessing possible environmental influences on the energy dynamics of these two spawning contingents that are managed as one stock complex.

## 1 INTRODUCTION

Atlantic herring *Clupea harengus* L. 1758 (hereafter herring), a commercially valuable species and important prey item for larger fish, birds and marine mammals, is currently at low population levels in the US and Canadian waters of the northwest Atlantic Ocean (Boyce et al., 2019; Kanwit & Libby, 2008; NOAA Fisheries, 2025). Efforts to rebuild the US herring fishery are complicated by the ongoing (12‐year) failure of the stock to produce a successful year class. Reasons for the recent sequence of poor recruitments (four of the lowest estimates in the history of the 60‐year time series occurring in the last 12 years) are not clear (NOAA Fisheries, 2025). During this period of low stock productivity, estimates of herring energy density (ED; kJ g^−1^ wet weight) are much lower than those from prior decades (Wuenschel et al., 2024), although the historical values rely on limited samples (Lawson et al., 1998; Steimle & Terranova, 1985). A decline in ED from the few historical records suggests that herring today may be in comparably worse energetic condition. The potentially poor current physiological health of herring could have ramifications for herring survival and reproduction as well as the species that prey on them.

In addition to long‐term changes in the ED of forage fishes over decades and/or productivity regimes, it can also vary substantially over smaller scales of space and time (i.e. seasonal, annual, regional) (Favreau et al., 2025; Renkawitz et al., 2015; Schismenou et al., 2024; Wuenschel et al., 2024). Intra‐annual ED variation is especially apparent in sexually mature herring, which are capital spawners and amass fat reserves during the feeding period to finance the energetic costs of reproduction, migration and overwintering (Kenyon et al., 2022; McBride et al., 2015; Murua & Saborido‐Rey, 2003; Sundby et al., 2016). The impact of these seasonal processes should be taken into account when interpreting the energetic condition of herring and evaluating temporal trends (dos Santos Schmidt et al., 2017; Hislop et al., 1991). The herring energetic cycle in the northwest Atlantic, as in other regions, can be particularly complex to untangle due to spatial overlap of multiple spawning components that can be energetically out of phase with one another (Daan et al., 1990; Wuenschel & Deroba, 2019). Spawning in this area may occur from April to November, but by convention, herring are classified into spring and fall contingents (Melvin et al., 2009). While the binary classification used in this region (and elsewhere) is useful and has a genetic basis (Fuentes‐Pardo et al., 2024), in reality, spawning in the northwest Atlantic Ocean occurs in waves that start in the spring and continue through the fall (Lambert, 1987; Sinclair & Tremblay, 1984). Because individuals mature and release a single cohort of eggs/milt per year, even minor reproductive timing differences (e.g. weeks) can lead to substantial ED variability (McBride et al., 2015; Murua & Saborido‐Rey, 2003). For example, in Norwegian spring‐spawning herring, gonad mass just prior to spawning averages 19% of the body mass (Varpe et al., 2005). Therefore, failure to account for time of year and spawning seasonality will increase the amount of unexplained variation in energetic condition (Wuenschel et al., 2024).

The complex array of herring life‐history tactics—such as differences in spawning seasonality, migratory behaviour and reproductive investment—is well‐documented in many regions (Bekkevold et al., 2023; dos Santos Schmidt et al., 2021; Mueller et al., 2023). However, for US Gulf of Maine and Georges Bank herring, investigations of the energy allocation patterns of the co‐occurring spawning contingents (which are managed as a single stock) are limited (Bradford & Stephenson, 1992; NOAA Fisheries, 2025; Wuenschel & Deroba, 2019). Considering the variable reproductive investment linked to body condition reported for other populations (Kennedy et al., 2011; Kurita et al., 2003; Ma et al., 1998; van Damme et al., 2009), knowledge of the energetic status of US herring could provide important context about stock dynamics to resource managers. This understanding is particularly critical amid the accelerated ecosystem change in the Gulf of Maine (Mills et al., 2024; Pershing et al., 2015) and given that unfavourable environmental conditions also have effects on herring fecundity that can span multiple seasons (dos Santos Schmidt et al., 2017). Further in‐depth investigations into herring ED are also necessary to evaluate if the recently reported lower ED (Wuenschel et al., 2024) indicates a true decline or is an artefact of non‐representative sampling.

Because of life‐history driven variation and substantial seasonal cycles, the full extent of this intra‐annual ED variability of US herring has been unresolved. Most historical information on US herring ED comes from studies that had a broad taxonomic focus and consequently did not achieve the sample size or temporal scale to accurately and precisely characterize the intra‐annual ED changes (Lawson et al., 1998; Steimle & Terranova, 1985; Wuenschel et al., 2024). For example, the estimate provided by Steimle and Terranova (1985) appears to be derived from just four individual herring collected at unknown points in the intra‐annual ED cycle of herring. Herring ED reported in Lawson et al. (1998) was estimated from 26 to 40 individuals primarily caught in October by the commercial fishery operating in nearshore east Newfoundland between 1991 and 1995. The herring in this area, during this time period, were predominately spring spawners (Melvin et al., 2009). Therefore, it is possible that the Lawson et al. (1998) estimate represents spawning‐recovered fish that completed depositing large stores of lipids in preparation for winter (Sundby et al., 2016). The ED reported by Wuenschel et al. (2024) evaluated samples collected during the spring (March–May) and fall (September–November), potentially excluding herring at peak ED, which for some stocks occurs near the summer solstice, when light‐related constraints on foraging subside (Kenyon et al., 2022; Varpe & Fiksen, 2010). Hence, to better explain the intra‐annual ED variation of US herring and facilitate direct comparisons to historical values, energetic condition measurements should be integrated with reproductive status.

In this study, we explored the seasonal variation in ED of spring‐ and fall‐spawning herring contingents in the northwest Atlantic Ocean. To enable large‐scale estimation of ED from percentage dry weight (%DW), we developed predictive relationships using proximate composition analysis (PCA)‐measured somatic and gonad ED. Quantification of the somatic and reproductive energetic condition of herring over a broad spatiotemporal scale allowed us to model ED in relation to day of year, sex and spawning contingent, and examine lipid and protein composition. A comprehensive understanding of variation in ED (or energy allocation) due to spawning seasonality is required to evaluate long‐term trends in herring ED and identify potential environmental influences on herring.

## 2 MATERIALS AND METHODS

### 2.1 Ethics statement

Sampling and animal handling protocols were carried out in compliance with the US National Marine Fisheries Service Institutional Animal Care and Use Policy (NMFS Procedure 04–112‐01) and Scientific Research Permits DA17‐008 SRP, DA18‐003 SRP and DA19‐003 SRP.

### 2.2 Field collection

Samples (*N* = 1104) were obtained from fishery‐independent and ‐dependent sources that encountered herring at different times and locations in the northwest Atlantic Ocean between March 2021 and May 2023 (Table 1 and Figure S1). When available, herring were selected in parallel with other existing herring sample requests. We sought to collect fish from as broad a spatiotemporal range as possible without negatively impacting survey sampling or monitoring of fishery operations. Given our objective of quantifying energetic differences between spawning contingents, when possible we sampled both sexes across a range of sizes and maturity stages from each tow. Fish were sealed in plastic bags, frozen at‐sea and transported to a laboratory freezer (−17°C) at the University of Massachusetts in Dartmouth, MA.

**TABLE 1:** Description of study sample sources: Northeast Fisheries Science Center Bottom Trawl Survey (BTS) and Northern Shrimp Survey (NSS), Massachusetts Division of Marine Fisheries Trawl Survey (MDMF) and Northeast Fisheries Observer Program (NEFOP).

Source	*n*	Operating period	Operation area	Years	Design	Gear
BTS	779	Spring (March–May) Fall (September–October)	Gulf of Maine to Cape Hatteras, North Carolina	2021–2023	Stratified random	3‐bridle, 4‐seam survey bottom trawl, rockhopper sweep, headrope height = 2.7–4.7 m
NSS	130	Summer (July–August)	Gulf of Maine	2021–2022	Stratified random	4‐seam modified commercial shrimp trawl
MDMF	124	Spring (May) Fall (September)	Territorial waters of Massachusetts	2021–2022	Stratified random	2‐seam otter trawl, rubber disc sweep
NEFOP	71	Year‐round	Gulf of Maine, Georges Bank, Southern New England, location information is proprietary	2021–2023	Commercial Fishing	Bottom trawl

### 2.3 Sample processing

Frozen herring (still in plastic bags) were transferred to refrigeration overnight to thaw. Specimens with visible injury (e.g. puncture wounds, scale damage, missing flesh) were omitted. Thawed fish were patted dry, measured for fork length (FL; ±1 mm), placed in a pre‐weighed aluminium pan, weighed (wet weight; ±0.01 g) and dissected in the pan to prevent fluid loss during processing. Sagittal otoliths were removed to determine age in years (by the lead herring age reader at the Northeast Fisheries Science Center [NEFSC]; see Section S1). During age reading, the presence/absence of false annuli was noted to assist with spawning contingent classification discussed below.

Sex (male, female or unknown) and maturity (immature, developing, ripe, ripe and running, spent or resting) were determined macroscopically following NEFSC criteria (Burnett et al., 1989). The gonad was extracted and placed in a pre‐weighed nylon biopsy bag (Fisherbrand®) and weighed (±0.001 g). The remaining herring body and viscera were analysed together and are referred to as the soma or somatic tissue. Gonadosomatic index (GSI; %) was calculated as:

$$\mathrm{GSI}=100\times \left(\frac{\text{gonad}\;\mathrm{wet}\;\text{weight}}{\mathrm{wet}\;\text{weight}-\text{gonad}\;\mathrm{wet}\;\text{weight}}\right)$$

For female ovaries in the developing or ripe stages, oocyte diameters of the advancing cohort were measured using approximately a 1 cm^3^ portion of ovarian tissue preserved in 10% neutral‐buffered formalin. To inform spawning contingent assignment for this subset of females, mean oocyte diameter, a precise and accurate maturation criterion (Óskarsson et al., 2002), was estimated using computer‐aided image analysis (see Thorsen and Kjesbu (2001) and McElroy et al. (2013) for more detail).

Herring somatic tissue (in pans) and gonads (in bags) were dried to a constant weight at 60°C. Following a minimum initial drying period of 7 days, samples were weighed every 24 h until a stable (minimum) weight was achieved (successive somatic tissue and gonad measurements differed by less than 0.1 g and 0.01 g, respectively). The %DW was calculated as:

$$\%\mathrm{DW}=100\times \left(\frac{\text{somatic}\;\mathrm{dry}\;\text{weight}+\text{gonad}\;\mathrm{dry}\;\text{weight}}{\mathrm{wet}\;\text{weight}}\right)$$

Somatic and gonad %DW were also calculated for each specimen. A subset of somatic tissue and gonad samples was chosen for ED determination through PCA (described below) to ensure the full range of observed sex, maturity stage, and %DW combinations were used to establish predictive %DW–ED linear regressions.

PCA followed prior studies (Morley et al., 2012; Wuenschel et al., 2024). The lipid weight of each component was determined using a Soxhlet extraction apparatus using petroleum ether as the solvent. Dried somatic tissue was placed into individual, pre‐weighed Alundum thimbles (100 cm in length, 3.5 cm in diameter). Larger individuals were divided into multiple thimbles and weights were re‐aggregated. All samples were weighed (±0.001 g, thimbles or dried gonad‐filled bags) prior to a 3 h extraction period. The extraction period was informed by Bean (2020), who observed complete lipid extraction (no weight change) for samples of similar composition and weight after 3 h. Following extraction, sample‐filled thimbles or bags were dried overnight at 60°C and weighed the next day to determine the lean (lipid‐free) dry mass. The lipid weight of the sample was calculated as the difference between the pre‐ and post‐extraction weights. Percentage lipid of each individual was calculated as:

$$\text{Percentage lipid}=100\times \left(\frac{\text{somatic lipid weight}+\text{gonad lipid weight}}{\mathrm{wet}\;\text{weight}}\right)$$

Somatic and gonad percentage lipid were also calculated for each specimen selected for PCA.

To estimate protein and ash weights, the lipid‐free somatic tissue samples (still in thimbles) and the lipid‐free gonad samples (after being placed into a pre‐weighed, covered crucible) were combusted in a muffle furnace at 600°C for 3 h to burn off any remaining organic matter. Some gonad tissue adhered to the bag mesh as it dried, therefore the entire gonad‐filled bag was burned and the bag weight was subtracted from the pre‐burn weight (pilot trials indicated biopsy bags completely combust at 600°C). Following combustion, somatic tissue and gonad samples were allowed to cool to ambient temperature, re‐dried overnight at 60°C and weighed 24 h later to determine protein and ash weights. The protein weight of each sample was the difference between the pre‐ and post‐burn weights (minus the bag weight, in the case of gonads). As in previous studies, combusted lipid‐free material was assumed to be protein weight and carbohydrate weight was ignored as it is a negligible component of fish tissue (Craig, 1977; Henken et al., 1986). The ash weight of each sample is the post‐burn weight of the sample (minus the pre‐weighed thimble/crucible). Percentage protein of each individual was calculated as:

$$\text{Percentage protein}=100\times \left(\frac{\text{somatic protein weight}+\text{gonad protein weight}}{\mathrm{wet}\;\text{weight}}\right)$$

Somatic and gonad percentage protein were also calculated for each specimen.

### 2.4 Energy estimation

Somatic and gonad energies were calculated by multiplying the lipid and protein weights by their respective energy equivalent values (39.565 kJ g^−1^ lipid and 23.64 kJ g^−1^ protein; Henken et al., 1986). The EDs of the gonad, soma and whole fish (combined soma and gonad) were then calculated by dividing the respective energies by their wet weight. We used the somatic and gonad ED values to create separate regressions for somatic tissue, gonad and whole fish to predict ED from %DW. For the gonad ED regression, we evaluated differences between sexes using ANCOVA. These regressions were used to estimate ED for all samples (gonad, soma or whole body) using their respective %DW. For consistency, in subsequent analyses we used predicted ED values for all samples. This approach allowed us to avoid some samples having an additional source of variation from the PCA measurement process.

When ovarian tissue was subsampled for oocyte measurement, PCA‐derived weights were scaled to the total gonad wet weight by dividing by the proportion of remaining tissue mass [total gonad wet weight/(total gonad wet weight – subsample weight)]. Immature fish had small gonads that did not contain enough material to reliably determine composition (minimum dry weight <1 g) with the gravimetric method used. Thus, variability in the %DW dramatically increased below this threshold as minute changes in weight represent a large proportion of the total sample weight. Given their small mass, gonad energy below this threshold was considered to be inconsequential in relation to the total fish energy, and as a result was assumed to be zero kJ.

### 2.5 Spawning contingent classification

Sexually mature herring were assigned to spring‐ or fall‐spawning groups using the macroscopic maturity determination, GSI, mean oocyte diameter, otolith false annulus presence/absence and the date of capture (Table 2). Mature herring not meeting the criteria (for either spring or fall) were assigned as having an unknown spawning group. Spawning group was not assigned to immature individuals that had not yet begun maturation and these fish were considered a separate category because their energy allocation patterns are different from mature individuals (Mr̊rtensson et al., 1996). By treating immature fish as a separate group, we sought to minimize the potential for a confounding effect from individuals without reproductive energetic demands. At certain times of the year, it is difficult to differentiate between spring and fall spawners using only macroscopic stage (e.g. developing spring spawners and early‐developing fall spawners caught in May). To provide additional resolution in months when both spawning contingents can be in the same macroscopic stage, we used GSI, mean oocyte diameter (when available) and otolith false annulus presence/absence to refine contingent classification (Table 2). Classification on the basis of false annulus presence was limited to age‐3 fish, as prior work has shown nearly all age‐3 spring spawners had a false annulus (90%; 140/154), compared to only 8% of fall spawners (9/108) (Warren, 2025). These additional criteria have been used in other studies to distinguish between spawning contingents: GSI (McPherson, 2010; McQuinn, 1989), mean oocyte diameter (McPherson, 2010) and false annulus presence/absence (Einarsson, 1951; Penttila & Dery, 1988).

**TABLE 2:** Monthly classification of herring spawning contingents based on macroscopic maturity stage.

Maturity stage	Month of capture									
	Jan	Feb	Mar	Apr	May	Jul	Aug	Sep	Oct	Nov
Immature	–	–	–	–	–	–	–	–	–	–
Developing	Spring	Spring	Spring	Spring	Spring Fall^a^	Fall	Fall	Fall	Fall Spring^b^	Fall Spring^b^
Ripe	Spring	Spring	Spring	Spring	Spring	Fall	Fall	Fall	Fall	Fall
Spent	Fall	Fall	Spring	Spring	Spring	Spring	Fall	Fall	Fall	Fall
Resting	Unk Spring^c^ Fall^d^	Unk Spring^c^ Fall^d^	Fall	Fall	Unk Spring^c^ Fall^d^	Spring	Spring	Unk Spring^c^ Fall^d^	Unk Spring^c^ Fall^d^	Unk Spring^c^ Fall^d^

*Note*: In some months (grey‐shaded cells), developing and resting stages could be either spring‐ or fall‐spawning individuals, so gonadosomatic index (GSI), false annulus presence/absence and mean oocyte diameter (when available) in conjunction with the date of capture were also used to classify spawning contingent. Mean oocyte diameter and GSI (of developing individuals) distributions at these times of the year (grey‐shaded cells) revealed distinct developmental trajectories and informed the contingent‐classification thresholds detailed in the table footnotes. No assignments were made for immature fish. Mature individuals were categorized as unknown (Unk) when they could not be assigned to either the spring or fall contingent.

^a^ Oocyte diameter is less than 400 μm or GSI is less than 2%.

^b^ Caught 17 Oct–30 Nov and oocyte diameter is less than 550 μm or GSI is less than 5%.

^c^ Age‐3 and false annulus present.

^d^ Age‐3 and false annulus absent.

### 2.6 Modelling intra‐annual energy density cycles

To model seasonal patterns in herring ED and assess differences between spawning contingents and sexes, we used a generalized additive model (GAM). This approach was chosen following data exploration, which revealed non‐linear effects of day of year on somatic ED. The effective degrees of freedom (edf >1) of the smooth terms subsequently confirmed this non‐linear relationship, supporting the use of a GAM over a generalized linear model. Samples collected from 2021 to 2023 were insufficient to fit annual GAMs so data were pooled across years to increase the number of available sampling dates. Although annual differences in somatic ED on a given day of year may occur, these shifts were assumed to be of lower magnitude than the large variation across day of year. In a study of intra‐ and inter‐annual variability in the fat content of North Sea herring from 2006 to 2020, the effect of day of year on fat content was similar between years (Kenyon et al., 2022).

Models were fit using the *gam* function within the R package *mgcv* (R Core Team, 2021; Wood, 2011), assuming a Gaussian distribution with an identity link. The restricted maximum likelihood estimation procedure was used to estimate smoothing parameters, with *mcgv* automatically determining the amount of smoothing. A cyclic cubic regression spline was specified for the day of year smoother to ensure the left‐ (beginning of the year) and right‐most (end of the year) were equivalent. Several models were fit to evaluate the contribution of day of year, sex and spawning contingent to the energetic condition of herring:

$$\text{Model}\;1-\text{Somatic}\;{\mathrm{ED}}_{i}=\alpha_{i}+f_{1}\left(\mathrm{Wet}\;{\text{Weight}}_{i}\right)+f_{2}\left(\mathrm{Day}\;{\text{of Year}}_{i}\;\right)+\varepsilon_{i}$$

$$\text{Model}\;2-\text{Somatic}\;{\mathrm{ED}}_{i}=\begin{array}{l} \alpha_{i}+\mathrm{Sex}+f_{1}\left(\mathrm{Wet}\;{\text{Weight}}_{i}\right)\\ +\;f_{3}\left(\mathrm{Day}\;{\text{of Year}}_{i}\times {\mathrm{Sex}}_{i}\right)+\varepsilon_{i} \end{array}$$

$$\text{Model}\;3-\text{Somatic}\;{\mathrm{ED}}_{i}=\begin{array}{l} \alpha_{i}+\text{Spawning Contingent}+f_{1}\left(\mathrm{Wet}\;{\text{Weight}}_{i}\right)\\ +f_{4}\left(\mathrm{Day}\;{\text{of Year}}_{i}\times {\text{Spawning Contingent}}_{i}\right)+\varepsilon_{i} \end{array}$$

where somatic ED is the energy density per gram of somatic wet weight (kJ g^−1^), $\alpha_{i}$ is the intercept, Sex and Spawning Contingent are nominal covariates, $f_{1}$ is the smooth function for wet weight (continuous variable; g), $f_{2}$ represents the smooth functions for day of year (continuous variable), $f_{3}$ represents the smooth functions for day of year that were allowed to vary by sex (categorical covariate; male, female or unknown), $f_{4}$ represents the smooth functions for day of year that were allowed vary by each spawning contingent (categorical covariate; fall, spring, unknown or immature) and $\varepsilon_{i}$ is the error term. For all models, somatic ED was selected as the response variable of interest because it was the most widely available energetic measure within our dataset and in sexually mature herring it has a strong intra‐annual signal which matches reproductive status (Kenyon et al., 2022).

Model diagnostics were performed to evaluate assumptions and potential model misspecification. Residuals were plotted versus fitted values and each covariate to evaluate the assumption of homogeneity. Normality of residuals was verified using a histogram and a quantile‐quantile plot. We used the *gam.check* and *concurvity* functions within *mcgv* to compare effective degrees of freedom with k for each covariate, evaluating model fit and assessing collinearity. GAM smoothers were visualized using the *visreg* and *ggplot2* R packages (Breheny & Burchett, 2017; Wickham, 2016).

## 3 RESULTS

### 3.1 Spawning contingent classification

**FIGURE 1:**
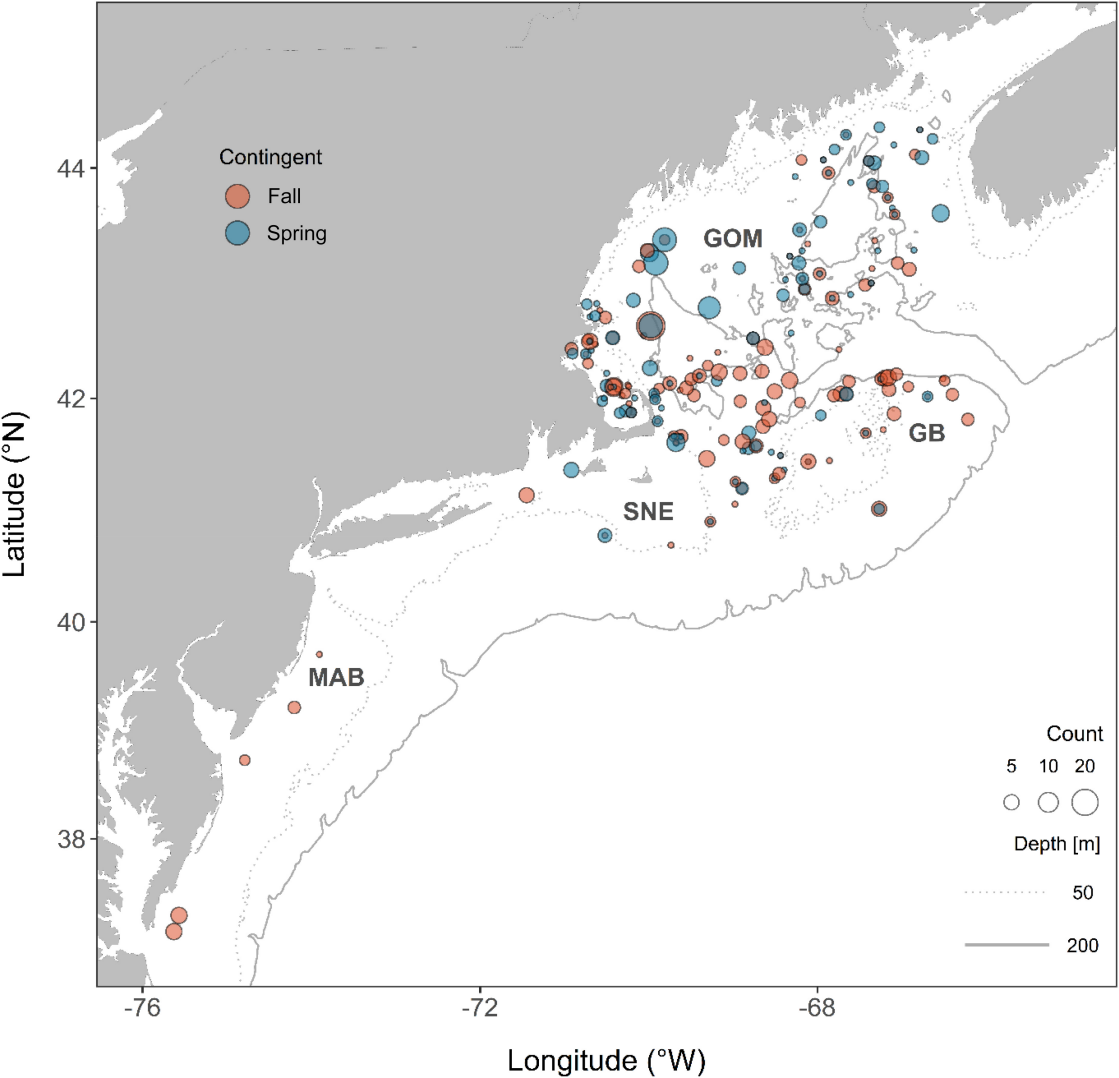
Map showing spring‐ (*n* = 251) and fall‐spawning (*n* = 340) herring sampling locations in the northwest Atlantic Ocean and number of individuals (size of symbol) collected 2021–2023 during the Bottom Trawl Survey, Northern Shrimp Survey and Massachusetts Division of Marine Fisheries Trawl Survey (see Figure S1 for source‐specific data). Northeast Fisheries Observer Program samples (spring spawners, *n* = 2; fall spawners, *n* = 22) are not shown. Grey lines indicate the 50 (dotted) and 200 (continuous) m isobaths. Abbreviated locations: Gulf of Maine (GOM), Georges Bank (GB), Southern New England (SNE) and Mid‐Atlantic Bight (MAB).

Between March 2021 and May 2023, we measured 1104 herring collected at different points of the year (10 of the 12 months). There was a considerable size overlap and comparable distribution between the study samples and all herring collected by the BTS during the same time period (Figure S2). Our sampling provided a broad range of lengths, weights and spawning conditions (Table 3). In the fall, herring were primarily collected on Georges Bank, the Gulf of Maine and the territorial waters of Massachusetts. In the spring, herring were encountered in the aforementioned locations, as well as Southern New England and the Mid‐Atlantic Bight. In the summer, most samples came from the Gulf of Maine (Figure S1). Spawning contingent assignment of these specimens resulted in 362 fall‐spawning and 253 spring‐spawning herring. Spring spawners were encountered more frequently at higher latitudes, while the majority of fall spawners were collected on the northern edge of Georges Bank (Figure 1). The remaining specimens were either immature (*n* = 386) or assigned to the unknown spawning season group (*n* = 103).

**TABLE 3:** Summary of the sample sizes, means and ranges for fork length, total wet weight and gonad wet weight of the study samples by source.

Source	*n*	Mean FL (mm)	FL range (mm)	Mean Total weight (g)	Total weight range (g)	Mean gonad weight (g)	Gonad weight range (g)
BTS Fall 2021	121	221 (10)	197–275	106.65 (13.80)	74.67–168.07	3.382 (6.265)	0.096–32.459
BTS Fall 2022	114	231 (23)	131–260	117.88 (25.53)	18.75–169.61	1.733 (3.874)	0.005–37.028
BTS Spring 2021	152	204 (22)	147–285	94.34 (36.29)	32.63–279.00	1.975 (6.942)	0.030–50.000
BTS Spring 2022	317	218 (22)	141–277	110.42 (35.90)	21.82–254.67	3.506 (5.563)	0.009–39.497
BTS Spring 2023	75	244 (7)	228–260	164.18 (21.37)	117.00–226.02	2.889 (3.265)	0.319–16.087
MDMF Fall 2021	43	206 (7)	195–218	87.62 (11.63)	71.95–119.78	2.134 (3.940)	0.071–21.389
MDMF Fall 2022	34	224 (12)	202–269	122.17 (27.78)	86.97–228.55	7.884 (9.921)	0.090–33.393
MDMF Spring 2022	47	191 (53)	55–274	88.96 (54.17)	0.83–255.42	0.930 (1.032)	0.001–5.353
NEFOP	71	231 (14)	174–257	120.90 (22.43)	54.44–184.07	1.971 (4.504)	0.152–25.690
NSS Summer 2021	5	225 (7)	220–236	136.57 (18.04)	113.40–154.25	7.644 (6.708)	2.329–19.197
NSS Summer 2022	125	228 (12)	208–267	121.68 (31.53)	79.06–206.16	3.255 (4.600)	0.098–25.285
Overall	1104	220 (24)	55–285	112.84 (35.57)	0.830–279.00	2.920 (5.560)	0.001–50.000

*Note*: Standard deviation is shown in parentheses.

Abbreviations: BTS, Bottom Trawl Survey; FL, fork length; MDMF, Massachusetts Division of Marine Fisheries Trawl Survey; NEFOP, Northeast Fisheries Observer Program; NSS, Northern Shrimp Survey.

### 3.2 Energy density estimates and sex‐specific allocation

**FIGURE 2:**
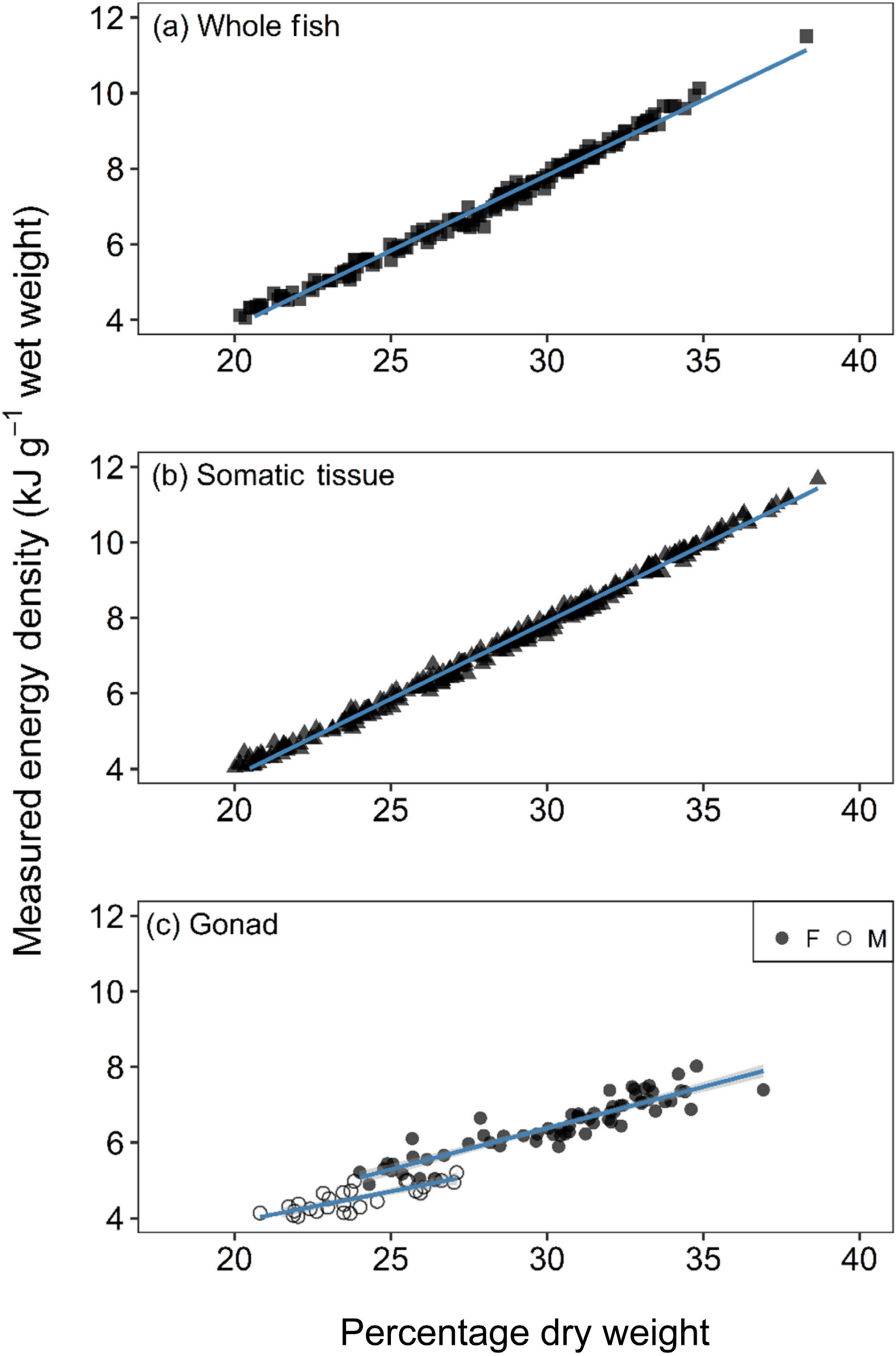
Relationship between percentage dry weight and measured energy density (ED) of (a) whole fish (■), (b) somatic tissue (▲), (c) ovary (●) and testes (○). ED was measured from proximate composition analysis. Regression statistics are shown in Table 4.

**FIGURE 3:**
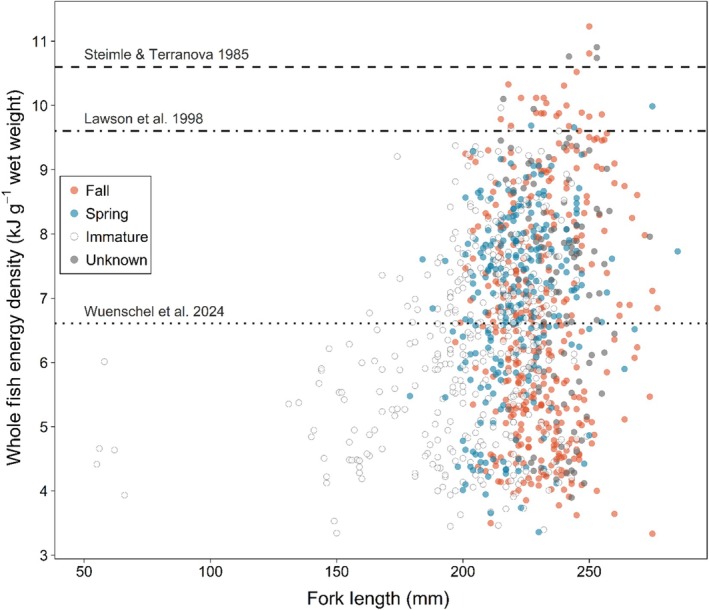
Predicted whole‐fish energy density (ED; *N* = 1104) by fork length. Horizontal lines indicate previously reported ED values: mean from Steimle and Terranova (1985; *n* = 4), mean from Lawson et al. (1998; *n* = 26–40) and median from Wuenschel et al. (2024; *n* = 634).

The respective %DW values of the somatic tissue, ovary, testes and whole fish were excellent, positive predictors of respective ED (Figure 2 and Table 4). Sex was a significant covariate in the %DW–gonad ED regression model, but only marginally increased the amount of variation explained by 1.6% (Tables S1 and S2). Fork length alone was not a good predictor of whole fish ED. There was a general trend of increasing variability in predicted whole fish ED for herring >200 mm in length (Figure 3). The majority of estimated whole‐fish ED values were below previous estimates from the 1980s and 1990s, but had the same median (6.61 kJ g^−1^ wet weight) as a more recent estimate from 2024 (Figure 3; Steimle & Terranova, 1985; Lawson et al., 1998; Wuenschel et al., 2024).

**TABLE 4:** Summary of linear regressions (parameter estimates ± SE) for energy density (ED) as a function of percentage dry weight (%DW) for somatic tissue, ovary, testes and whole fish.

Component	*n*	*a* ± SE	*b* ± SE	Adj. *r* ^2^	*p* value	%DW range
Somatic tissue	295	−4.19 ± 0.05	0.403 ± 0.002	0.994	<0.001	18.73–38.66
Ovary	65	0.03 ± 0.26	0.212 ± 0.009	0.961	<0.001	24.01–36.92
Testes	46	−0.54 ± 0.35	0.212 ± 0.009	0.961	<0.001	18.98–27.11
Whole fish	173	−4.13 ± 0.09	−0.399 ± 0.003	0.990	<0.001	20.18–38.30

*Note*: ED = *a* + *b* × %DW. Gonad ED only estimated for gonads with a dry weight greater than 1 g.

Abbreviation: SE, standard error.

**FIGURE 4:**
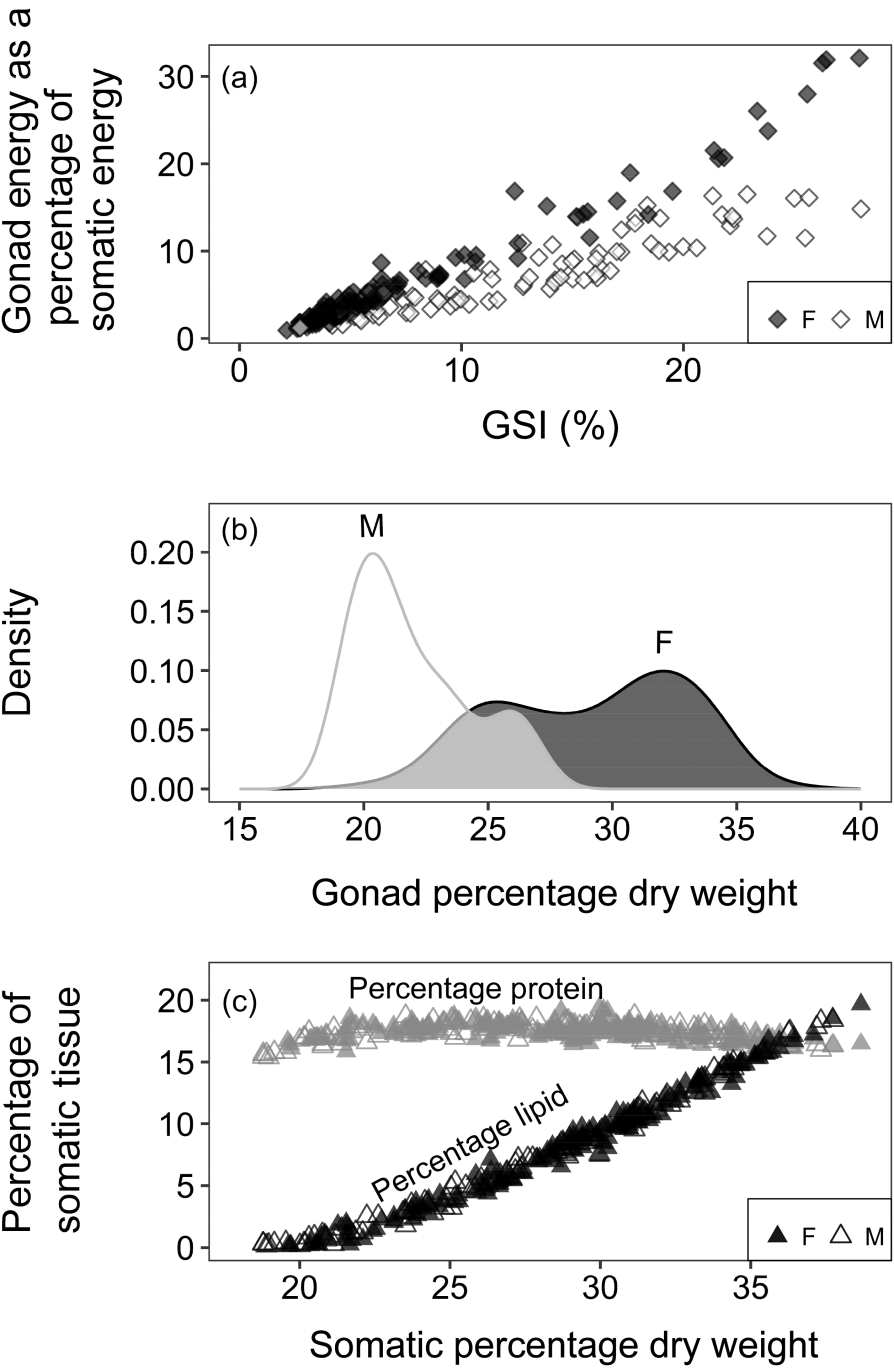
Sex‐specific energy allocation, gonad percentage dry weight (%DW) and somatic tissue composition. (a) Female (♦; *n* = 128) and male (♢; *n* = 82) and relationship between gonadosomatic index (GSI) and gonad energy as a proportion of somatic energy. Gonad and somatic energies were estimated using the regressions in Table 4. (b) Smoothed density functions of gonad %DW for females (filled; *n* = 103) and males (transparent; *n* = 77). (c) The relationship between somatic %DW and somatic percentage lipid (black) and somatic percent protein (grey) for females (▲, *n* = 187) and males (△, *n* = 105). Samples with a gonad dry weight of less than 1 g are not included in plots (a) and (b).

The PCA of the gonads also indicated sex‐specific energetic differences. Females invested more energy in the gonad compared to males (Figure 4). In males with a GSI greater than 20%, the testes energy did not exceed 17% of the somatic energy and plateaued as GSI increased further. In contrast, female gonad energy continued to increase linearly at GSI >17% and ovary energy became a higher proportion of somatic energy at high GSI (>20%) (Figure 4a). The maximum observed %DW of the testes did not exceed 27.1%, while the ovaries routinely exceeded this value and reached a maximum %DW of 36.9% (Figure 4b). In both sexes, percentage somatic protein stayed consistent as %DW increased, remaining between 15.4% and 19.4%, while percentage somatic lipid increased linearly (Figure 4c). Changes in somatic lipid were the biggest driver of ED in herring.

### 3.3 Intra‐annual variation in somatic energy density

**FIGURE 5:**
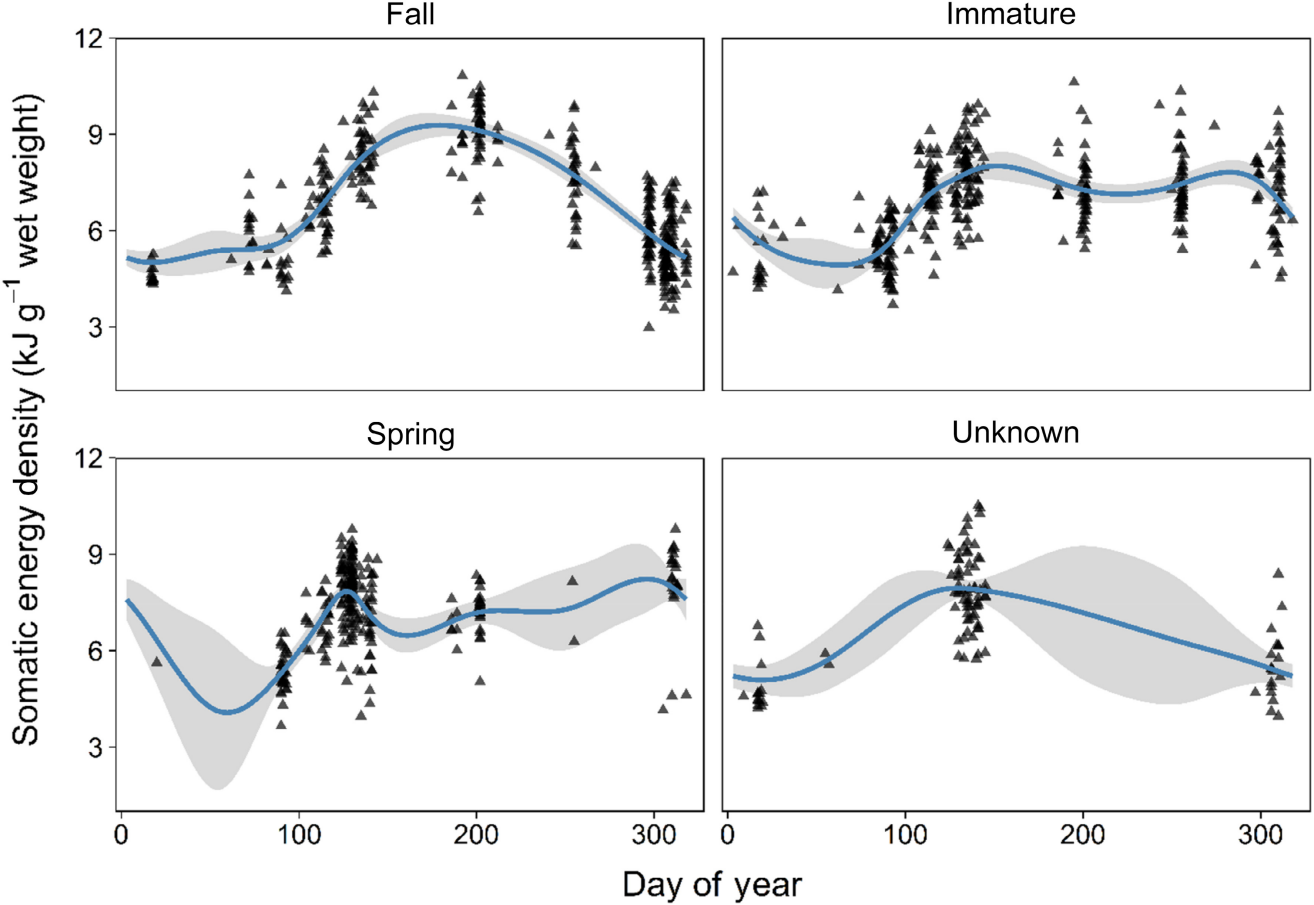
Model 3 (Table 6) predicted seasonal patterns in somatic energy density (ED) for fall‐spawning, spring‐spawning, unknown and immature herring. Individual points depict partial residuals and shaded regions represent the 95% confidence interval. Note the use of cyclic cubic regression splines that enforce continuity in predicted somatic ED at the start and end of the day of year range.

**FIGURE 6:**
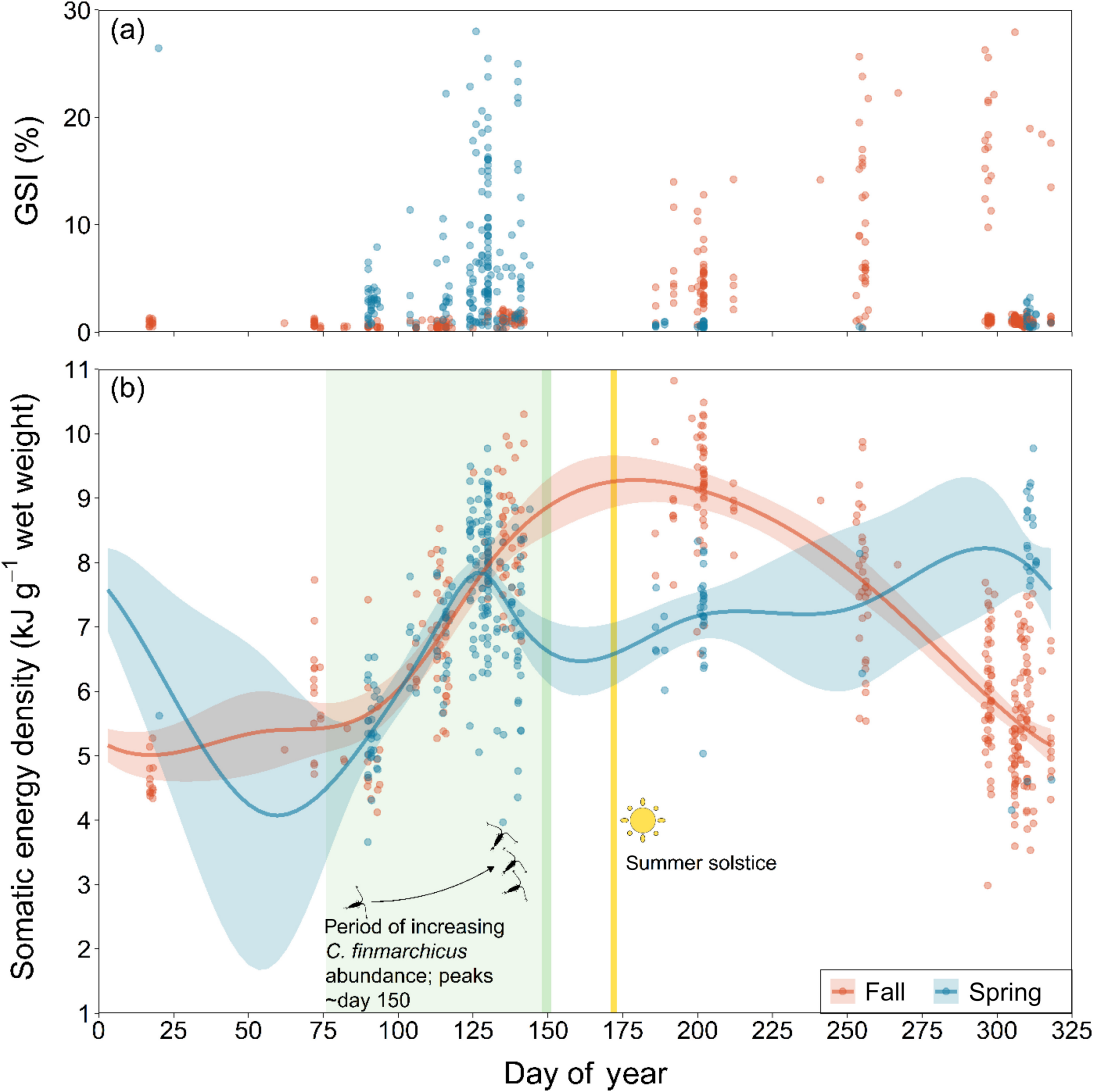
Seasonal dynamics of reproductive investment and somatic energy density (ED) in herring contingents. (a) Gonadosomatic index (GSI) by day of year for spring‐spawning (blue) and fall‐spawning (red) herring. (b) Model 3 (Table 6) predicted seasonal patterns in somatic ED. Individual points depict partial residuals and blue/red shaded regions represent the 95% confidence interval. Green area indicates time period in the western Gulf of Maine when increases in *Calanus finmarchicus* abundance are at their most rapid (Runge et al., 2025) and the yellow vertical line denotes the summer solstice. Use of cyclic cubic regression splines ensures continuity at the start and end of the day of year range.

GAMs were successfully fit and indicate that spawning contingent explained more of the variation in herring somatic ED than sex (Table 5). The inclusion of an interaction between the day of year smoother and spawning contingent (Model 3) increased the percentage of deviance explained (% dev) by 12.2% (from Model 1). In contrast, the inclusion of sex only increased the % dev by 1.3% (Model 2). The % dev differences between the models that use data subset by spawning contingent (Models 2a, 2b, 2c) also suggest that the impact of sex on day of year ED is minimal (Table 5). Model performance was strongly influenced by fall‐spawning contingent fish, as Model 2a, which excluded immature and spring‐spawning individuals, explained the most variation (74.4% % dev). Of the models that include the full dataset (Model 1, Model 2 and Model 3), Model 3 (with spawning contingent information and without sex) had the lowest AIC and indicated day of year ED pattern differences between spring and fall contingents (Tables 5 and 6, and Figures 5 and 6).

**TABLE 5:** Summary of GAMs fit to predict somatic tissue ED_*i*_. The additive terms, Akaike's information criterion (AIC) and % Dev are listed for each model.

Model	Data (*n*)	Additive terms	AIC	% dev
1	All (*n* = 1104)	$\alpha_{i}+f_{1}\left(\mathrm{Wet}\;{\text{Weight}}_{i}\right)+f_{2}\left(\mathrm{Day}\;{\text{of Year}}_{i}\;\right)+\varepsilon_{i}$	3444	52.4
2	All (*n* = 1104)	$\alpha_{i}+\mathrm{Sex}+f_{1}\left(\mathrm{Wet}\;{\text{Weight}}_{i}\right)+f_{3}\left(\mathrm{Day}\;{\text{of Year}}_{i}\times {\mathrm{Sex}}_{i}\right)+\varepsilon_{i}$	3431	53.7
2a	Fall (*n* = 362)		1002	74.4
2b	Spring (*n* = 253)		741	51.2
2c	Immature (*n* = 386)		1117	55.5
3	All (*n* = 1104)	$\alpha_{i}+\text{Spawning Contingent}+f_{1}\left(\mathrm{Wet}\;{\text{Weight}}_{i}\right)+f_{4}\left(\mathrm{Day}\;{\text{of Year}}_{i}\times \text{Spawning}\;{\text{Contingent}}_{i}\right)+\varepsilon_{i}$	3154	64.6

*Note*: See Table 6 and S3–S7 for GAM results. Sexually mature individuals that could not be assigned to a spawning group (unknown; *n* = 103) were not modelled separately.

**TABLE 6:** Generalized additive model (GAM) results for the best‐fitting model (Model 3) predicting herring somatic energy density.

Component	Term	Estimate	Std error	*t* value	*p* value
A. parametric coefficients	(Intercept)	6.78	0.07	104.25	<0.001
	Immature	0.03	0.10	0.27	0.7847
	Spring	0.11	0.12	0.92	0.3584
	Unknown	−0.15	0.23	−0.68	0.4984

Component	Term	edf	Ref. df	*F* value	*p* value
B. smooth terms	s(Wet weight)	3.71	4.70	42.00	<0.001
	s(Day of year):Fall	5.69	8.00	84.58	<0.001
	s(Day of year): Immature	6.34	8.00	36.43	<0.001
	s(Day of year):Spring	6.21	8.00	19.80	<0.001
	s(Day of year):Unknown	2.63	8.00	19.85	<0.001

*Note*: Adjusted R‐squared: 0.64, Deviance explained 64.6%. ‐REML: 1609.99, Scale est.: 0.99, *N*: 1104. The model includes both parametric (linear) and smooth (non‐linear) functions of covariates.

Model 3 predicted that all groups (spring‐spawning, fall‐spawning, immature and unknown) undergo a period of rapid ED increase after day 60 (1 March) (Figure 5). The rate of increase was similar, but the starting and ending points of this initial rise in ED varied by spawning contingent. Spring spawners appeared to begin this period of steep increase at lower ED, which only increased through day 127 (7 May) (Figures 5 and 6). In contrast, fall spawners had higher ED around day 60, which increased over a longer period and reached a higher maximum on day 178 (27 June). Spring‐spawning fish appear to have a secondary ED peak at day 295 (22 October) and enter winter with higher ED compared to fall spawners, which are near their annual minimum ED values (Figure 6). Immature herring ED increased in the spring and was consistent though fall before declining over winter (Figure 5). The sexually mature fish of unknown spawning season were clustered at certain times of year (when it is difficult to distinguish spring from fall spawners) and likely included individuals from both spawning contingents, confounding interpretation of seasonal ED patterns (Figure 5).

The relationship between somatic ED and GSI varied between and within spawning contingents (Figure 6). Within the spring spawners, Model 3 predicted that individuals spawning earlier in the season experienced a decline in somatic ED as GSI increased, whereas those spawning later showed a simultaneous increase in both metrics. The somatic ED of fall spawners declined as their GSI increased, though at a less steep rate than the decline predicted for spring spawners between day 0 and 50.

## 4 DISCUSSION

We documented a large amount of variation in the ED of herring in the northwest Atlantic Ocean, which is consistent with other studies on herring energetics (Kenyon et al., 2022; Slotte, 1999; Wuenschel et al., 2024). This finding was expected given the wide range of dates, sizes, spawning contingents and maturity stages examined (Table 3). In contrast to Wuenschel et al. (2024), we were able to evaluate the relative influence of sex, maturity and spawning contingent on ED. Inclusion of these covariates in the GAM improved model fits (more deviance explained) and thus provides insights into spawning contingent‐specific energy tactics.

The strong intra‐annual patterns in ED vary by spawning contingent, maturity (immature versus mature) and, to a lesser extent, sex (Figures 4 and 5). Although sex‐specific energy dynamics are present, ED variability attributable to sex was relatively minor compared to the variation due to day of year and spawning contingent (Table 5, and Figures 4 and 5). Pronounced seasonal cycles in energy have been reported for many species (Jansen et al., 2021; Kenyon et al., 2022; Vollenweider et al., 2011) and tend to be stronger in total spawners. For example, in winter flounder *Pseudopleuronectes americanus* (Walbaum 1792) the annual muscle energy cycle had a higher amplitude compared to batch‐spawning yellowtail *Myzopsetta ferruginea* (Storer, 1839) and summer flounder *Paralichthys dentatus* L. 1766 (Wuenschel et al., 2019). We also established predictive %DW–ED relationships (of the whole fish, somatic tissue and gonad; Table 4) and show that the large variation of % somatic lipid drove shifts in energetic condition (Favreau et al., 2025; Martin et al., 2017), while % somatic protein remained stable (Figure 4c). Similar stable protein and variable lipid dynamics occurred in Atlantic mackerel *Scomber scombrus* L. 1758 and other forage species (Jansen et al., 2021; Vollenweider et al., 2011). While high ED was observed in both sexes, female herring allocated larger amounts of energy as well as a larger proportion of their somatic reserves to reproduction (Figure 4a). These results, particularly our estimates of the seasonal herring ED cycle, provide important context for interpreting energy estimates from prior work and evaluating long‐term trends as well as assessing possible environmental influences on the energy dynamics of these two spawning contingents that are managed as one stock complex (NOAA Fisheries, 2025).

The contrasting intra‐annual somatic ED patterns for spring‐ and fall‐spawning herring are a consequence of reproductive timing differences between the two contingents. In the North Sea, Wood (1958) observed distinct fat content cycles for winter‐ and early‐autumn‐spawning contingents, which resemble the fall and spring spawner ED patterns identified in our study, respectively. The smooth maximum for day of year predicted somatic ED of fall spawners was higher compared to the initial ED peak of spring spawners (Figure 6). Previous work has shown that fall spawners generally have more somatic lipid at spawning compared to spring spawners (Bradford, 1993b); a difference attributed to the longer overlap between the gametogenesis of fall contingent fish and the feeding season (Bradford, 1993a; dos Santos Schmidt et al., 2021). Herring that are able to allocate energy directly to gametes during peak foraging conditions in the spring and summer may reduce energy losses associated with storage and remobilization, compared to individuals spawning early in the year, which undergo gonadal development during the winter and likely face greater metabolic costs related to energy storage and transfer (from soma to gonad) (Bradford, 1993a; dos Santos Schmidt et al., 2021).

Total spawners like herring are often associated with a strict capital breeding strategy; however, the seasonal somatic ED and GSI patterns of herring in the US‐managed stock potentially indicate flexibility in reproductive energy allocation. Over the protracted (April–November) spawning season, the temporal alignment of gonadal development with food availability varies at the individual‐level, suggesting that the position of herring along the capital–income breeding strategy continuum is not uniform (McBride et al., 2015; Schismenou et al., 2024). Herring spawning earlier in the season (April–May) occupy the extreme capital end of the spectrum, similar to other stocks (Kennedy et al., 2010; Slotte, 1999), with gonadal development primarily fuelled by prior‐year reserves. For individuals spawning mid‐season (June–August), gonadal development is more aligned with the seasonal productivity pulse, allowing these fish to partially offset reproductive costs with ingested energy. This apparent mixed capital‐income breeding strategy is supported by the observed simultaneous increase of somatic ED and GSI (Figure 6). For herring spawning later in the year (September–November), gonadal development is less aligned with peak food resources, and there is an increased reliance on stored somatic energy (though unlike early‐season spawners, these individuals draw from energy stored in the current year). While this is also indicative of a capital breeding strategy, the more gradual decline of somatic ED observed in these fall spawners suggest they may be able to reduce energy losses to some extent through feeding. Although some herring stock components certainly meet the criteria of extreme capital spawning, our results imply that spawning phenology influences the degree to which current‐year energy intake can offset the somatic costs of reproduction.

The GAM smooths for spring‐ and fall‐spawning herring show different somatic ED maximums in spring/summer due to contingent‐specific life‐history tactics. The lower initial peak somatic ED in spring herring could result from movement away from the feeding grounds (at a time when conditions for energy gain are optimal) to spawning areas where prey abundances may be lower (Figure 6). This interruption of the main feeding period is also thought to explain fat content differences observed in spring and autumn spawners in southwest Newfoundland (Parsons & Hodder, 1975). In addition to movement from prime feeding locations, there may also be reduced feeding activity with advancing gonadal development. In an analysis of fish feeding and maturity stages in the northwest Atlantic Ocean, herring increased feeding after spawning (Link & Burnett, 2001). Specifically, they found that the amount of prey in the stomach was significantly lower during the developing stage compared to the post‐spawning stages. In advanced‐developing herring with high GSI, the enlarged gonads likely limit gut capacity due to spatial constraints within the coelomic cavity (Weeks, 1996). This space limitation may explain why substantial feeding does not occur in the late stages of gonad maturation (Bradford, 1993a) or during the spawning migration (Parsons & Hodder, 1975; Slotte, 1999) despite the availability of abundant prey. This physical constraint on energy intake during peak prey abundance and light availability (from day 75–175) primarily affects spring spawners and may in part explain their inability to gain as much energy as fall spawners (Figure 6).

Spring spawners undergo gonadal development over winter (dos Santos Schmidt et al., 2021; McQuinn, 1989), which is illustrated by the steeper winter somatic tissue ED decline (from day 295–50 of following year). Despite ending the calendar year with higher ED (day 275–325), the somatic ED of spring spawners decreases to levels below or equivalent to fall spawners by the ensuing spring (day 50–100) (Figure 6). Spring spawners appear to finance these winter reproductive costs through stored energy reserves accrued from feeding opportunities after spawning that allow them to enter winter with higher ED compared to fall spawners. Wood (1958) also observed this secondary peak later in the year in the fat content of summer‐spawning North Sea herring. The different contingent‐specific intra‐annual ED patterns, in which spring spawner ED is higher than that of fall spawners after day 275, suggest that individuals spawning early in the year rely to a greater extent on stored energetic capital to fuel gonadal development over winter. While much of the energy accumulated prior to winter gets allocated to reproduction in spring spawners, it is well‐established that herring can downregulate their reproductive investment if conditions are unfavourable (dos Santos Schmidt et al., 2020; Kennedy et al., 2011; Kurita et al., 2003). Compared to fall spawners, some spring spawners in the Gulf of Maine may be more prone to fecundity downregulation because they rely to a greater extent on stored energy reserves and do not supplement with direct feeding to the same extent as fall‐spawning herring.

The late‐June timing of peak ED for fall spawners in this study was identical to the fat maximum identified in the North Sea herring stock by Kenyon et al. (2022). In the western Gulf of Maine, the abundance of *Calanus finmarchicus*, an important prey for herring, historically undergoes a rapid increase from mid‐March to late‐May, before peaking around day 150 (Runge et al., 2025). The GAM smooths indicate that the period of pronounced ED gains for both spawning contingents coincides with this large increase in *C. finmarchicus* abundance, although the timing of peak somatic ED relative to the peak abundance of *C. finmarchicus* is different for spring and fall spawners (Figure 6). While they are sympatric populations that share the same feeding grounds (McQuinn, 1997), the aforementioned behavioural (migration) or physical (enlarged gonad impeding consumption) constraints slow energy gains for spring spawners, leading to an earlier, lower peak somatic ED that precedes maximum *C. finmarchicus* abundance, whereas fall spawners continue to gain energy, reaching a later and higher initial somatic ED peak. Day length may also be influencing the observed ED patterns independent of prey abundance. In Norwegian spring‐spawning herring, food intake has been shown to increase with day of year until the summer solstice, with the easing of light‐related constraints on foraging playing a major role in the timing of the increase in body condition (Varpe & Fiksen, 2010). The observed slow increase of somatic ED from day 25 to day 80 in fall spawners that precedes the period of rapid increase may be indicative of these visual limits imposed by day length at that time of year (Figure 6).

While we obtained samples in 10 out of 12 months and our models correspond with the trends reported in other regions (Kenyon et al., 2022; Parsons & Hodder, 1975; Wood, 1958), the ability of the GAM to predict contingent‐specific energetic patterns could be improved by more observations during winter and summer, as well as more advanced contingent identification techniques. Model 2a, which only considered fall‐spawning herring, likely benefitted (highest % dev; Table 5) from more samples spread throughout the year and reproductive cycle as compared to models for other spawning contingents that had fewer and less evenly distributed observations. To incorporate additional samples at certain times of year, more detailed and precise classification methods may be necessary to allow for accurate assignment of spawning contingent when spring‐ and fall‐spawning fish cannot reliably be distinguished using macroscopic maturity classification (McPherson, 2010; McQuinn, 1989; Wood, 1958; Wuenschel & Deroba, 2019). Gonadal histology or recent genetic advancements that identified loci associated with spring‐ and fall‐spawning herring were beyond the scope of this study, but would allow for the differentiation between contingents at particular times of the year, like the fall, when the ability to classify resting fish who may either be spawning‐recovered or preparing‐to‐spawn (in the spring) is difficult or otherwise not possible (Kerr et al., 2019; Lamichhaney et al., 2017; Wuenschel & Deroba, 2019).

Although recent research on the energetic condition of forage fish in the northwest Atlantic reported nearly a 50% decline in herring ED compared to prior estimates from the 1980s and 1990s (Lawson et al., 1998; Steimle & Terranova, 1985; Wuenschel et al., 2024), our results suggest this discrepancy may be attributable in part to sampling different times of the annual cycle we report. The wide range of observed ED is likely the result of reproductive plasticity that governs intra‐annual energy intake and allocation patterns which are out of phase to varying degrees throughout the annual cycle (Figure 5). Over the course of a year, herring ED decreased by half in the winter from the annual maximum in late spring/summer. Some of the ED maximums in our dataset, generally seen in herring collected on the summer NSS survey, did overlap with the estimates produced by Steimle and Terranova (1985) and Lawson et al. (1998) (Figure 3). Given the ED differences between herring caught on the spring and fall BTS (sample source for Wuenschel et al. (2024)) and the sampling regimens of the two earlier studies, we caution against direct comparisons to prior studies that sampled different times of the year (Figure 5). Unfortunately, the limited data from earlier studies is inadequate to confidently evaluate long‐term trends in herring ED and its relation to recruitment failures or the rapid climate change in the Gulf of Maine (Mills et al., 2024; NOAA Fisheries, 2025; Pershing et al., 2015).

By determining the macroscopic maturity stage, reproductive and somatic energy of herring throughout much of the year, we were able to model the day of year energetic patterns for spring‐ and fall‐spawning as well as immature herring, explaining more variation in ED than GAMs based on size, depth, season and location (Wuenschel et al., 2024). Doing so helps to partition out a major source of energetic variability within the regional herring stock complex and facilitates the comparison of different life‐history strategies and stages (Schismenou et al., 2024). The consideration of contingent and other factors, such as day of year, is important for both continued energetic monitoring and bioenergetic modelling efforts. Model 3 in this study predicts a larger discrepancy between contingents during the fall (when one group has just spawned and the other has recovered from spawning). If monitoring programs cannot account for reproductive stage and spawning contingent, it may be prudent to restrict any analysis of long‐term trends (in the study area) to samples collected in spring, when reproductive and contingent effects on herring ED are reduced (Figure 6). In addition, assessments and energetics models for species that prey on herring (and incorporate herring energy) will need to consider seasonal variation in herring ED, particularly when consumption is focused in certain seasons.

Herring are vulnerable to climate change, with concerns related to early life‐history and a potential northward distribution shift (Hare et al., 2016). Recent regional analysis of fishery independent survey and environmental data (Mills et al., 2024) indicated herring shifted distribution (but not enough to avoid warming) and decreased in length at all ages >2. This inability of herring to track optimal conditions likely incurs an energetic cost, suggesting that with continued monitoring it may be possible to empirically link energetic condition with environmental drivers like temperature or primary production and thus better understand the underlying physiological mechanisms that influence stock productivity. Beyond direct temperature effects, changes in spatial distribution may mirror changes in their preferred prey to maintain energy intake and condition. Such a distributional shift could be a response to declining productivity in the historic/current range. Net energy gain from the environment is theorized to decline at greater distances from a population's geographic centre (Wingfield et al., 2015), implying that with warming, herring energetic condition throughout the southern extent of their range may deteriorate as the distribution shifts poleward. Therefore, tracking herring ED could serve as an early warning signal for detecting these climate‐driven range shifts.

Our results enhance ongoing monitoring by providing the necessary context to evaluate condition measurements, which are complicated in the US‐managed herring stock by variation due to temporal differences in spawning. In light of its current stock status, recent decrease in recruits per spawner (Mills et al., 2024), importance as a prey species and commercial value, herring ED can be a valuable indicator in a shifting environment. The recent disappearance of more than 10 billion snow crab (*Chionoecetes opilio*) in the eastern Bering Sea, a mass starvation event connected to increased caloric demands during a marine heatwave, is a stark reminder of the necessity of species‐level monitoring to detect and respond to the effects of new ecosystem conditions (Szuwalski et al., 2023). A mechanistic understanding of energy dynamics in herring will improve ecosystem and predation models, and inform assessments of these important forage fish and the species that rely on them.

## AUTHOR CONTRIBUTIONS

Ideas: all authors. Data generation: J.B.W. Data analysis: J.B.W. and M.J.W. Funding: K.O. and M.J.W. Supervision: M.J.W. and K.O. Methodology: all authors. Project administration, visualization, writing – original draft: J.B.W. Manuscript preparation: all authors.

## FUNDING INFORMATION

This work was supported in part through funding from the NOAA Cooperative Institute for the North Atlantic Region grant NA19‐OAR‐4320074.

## CONFLICT OF INTEREST STATEMENT

The authors declare that there is no conflict of interest.

## Supporting information


**DATA S1.** Supporting Information.

## Data Availability

The data that support the findings of this study are available from the corresponding author upon reasonable request.
