# Supplementary material for: Intra‐annual energy density cycles of spring‐ and fall‐spawning Atlantic herring Clupea harengus reveal different reproductive allocation tactics
Source: J Fish Biol. 2026 Mar 17;109(1):220–35. doi: 10.1111/jfb.70400 (PMC13397281; doi:10.1111/jfb.70400)
Supplement: Supplementary file 1 — DATA S1. Supporting Information. [file JFB-109-220-s001.docx]

**Supplementary Material**

*Section 1: Otolith extraction, storage, and age reading*

Otolith preparation followed the NEFSC Age & Growth lab herring protocol. Extracted sagittal otoliths were stored in vials with tap water for 24 h. The next day, otoliths were patted dry, stored in clean vials, allowed to further dry in a fume hood for a minimum of a week before each pair was embedded (distal otolith surface up) using a xylene-based rapid drying medium (Epredia^TM^ Cytoseal^TM^ XYL) in a circular depression on a black molded tray (10” x 5”, 1/16” depression, Exact Dispensing Systems). Annuli were viewed under reflected light and enumerated using a dissecting scope (20x magnification).





Figure S1. Map showing herring sampling locations in the northwest Atlantic Ocean and number of individuals (size of symbol) collected 2021–2023 during BTS, NSS, and MDMF. NEFOP sampling locations are proprietary and not shown. The grey lines indicate the 50 (dotted) and 200 (continuous) m isobaths. Abbreviated locations: Gulf of Maine (GOM), Georges Bank (GB), Southern New England (SNE), and Mid-Atlantic Bight (MAB). For a delineation of the territorial waters of Massachusetts, please refer to: <https://www.neamap.net/madmf-bottom-trawl/> .





Figure S2. Smoothed density functions of herring fork length (mm) for study samples (yellow; *N* = 1104) and Northeast Fisheries Science Center bottom trawl survey (2021–2023) samples (gray; *N* = 12,097). While Kolmogorov-Smirnov and Wilcoxon Rank-Sum tests both indicate statistically significant differences (p <0.05), the visual evidence and small effect size (0.024) suggests limited biological difference between the sample source distributions. At large sample sizes these statistical tests have increased power and are likely able to detect very small differences.

Table S1. Summary of linear regression for gonad energy density (kJ/g wet weight) as a function of gonad percent dry weight (%DW). Gonads with a dry weight of less than a gram were excluded from analysis.

|  | **Estimate** | **Std Error** | **t value** | **p-value** |
| --- | --- | --- | --- | --- |
| (Intercept) | -1.48 | 0.16 | -9.02 | <0.001 |
| Gonad %DW | 0.259 | 0.006 | 43.47 | <0.001 |
| Residual standard error: 0.30 on 109 degrees of freedom | | | | |
| Multiple R-squared: 0.95, Adjusted R-squared: 0.95 | | | | |
| F-statistic: 1890 on 109 and 1 DF, p-value: < 0.001 | | | | |

Table S2. Summary of linear regression for gonad energy density (kJ/g wet weight) as a function of percent gonad dry weight (%DW) and sex. Gonads with a dry weight of less than one gram were excluded from analysis.

|  | **Estimate** | **Std Error** | **t value** | **p-value** |  |
| --- | --- | --- | --- | --- | --- |
| (Intercept) | 0.03 | 0.26 | 0.12 | 0.904 |  |
| Gonad %DW | 0.212 | 0.009 | 24.57 | <0.001 |  |
| SEX_M | -0.57 | 0.09 | -6.71 | <0.001 |  |
| Residual standard error: 0.26 on 108 degrees of freedom | | | | | |
| Multiple R-squared: 0.96, Adjusted R-squared: 0.96 | | | | | |
| F-statistic: 1350 on 108 and 2 DF, p-value: < 0.001 | | | | | |

Table S3. Model 1 GAM results.

| **Component** | **Term** | **Estimate** | **Std Error** | **t-value** | **p-value** |  |
| --- | --- | --- | --- | --- | --- | --- |
| A. parametric coefficients | (Intercept) | 6.68 | 0.03 | 194.13 | <0.001 |  |
| **Component** | **Term** | **edf** | **Ref. df** | **F-value** | **p-value** |  |
| B. smooth terms | s(Wet weight) | 2.50 | 3.21 | 86.47 | <0.001 |  |
|  | s(Day of year) | 6.86 | 8.00 | 91.33 | <0.001 |  |
| Adjusted R-squared: 0.52, Deviance explained 52.4% | | | | | | |
| -REML : 1733.10, Scale est: 1.31, N: 1104 | | | | | | |

Table S4. Model 2 GAM results.

| **Component** | **Term** | **Estimate** | **Std Error** | **t-value** | **p-value** |
| --- | --- | --- | --- | --- | --- |
| A. parametric coefficients | (Intercept) | 6.71 | 0.05 | 137.06 | <0.001 |
|  | SEX_Unknown | -0.18 | 0.30 | -0.60 | 0.549 |
|  | SEX_M | -0.05 | 0.07 | -0.72 | 0.471 |
| **Component** | **Term** | **edf** | **Ref. df** | **F-value** | **p-value** |
| B. smooth terms | s(Wet weight) | 2.30 | 2.98 | 66.31 | <0.001 |
|  | s(Day of year):SEX_F | 6.34 | 8.00 | 56.88 | <0.001 |
|  | s(Day of year):SEX_Unknown | 0.80 | 5.00 | 0.31 | 0.143 |
|  | s(Day of year):SEX_M | 6.02 | 8.00 | 40.83 | <0.001 |
| Adjusted R-squared: 0.53, Deviance explained 53.7% | | | | | |
| -REML : 1732.07, Scale est: 1.28, N: 1104 | | | | | |

Table S5. Model 2a GAM results.

| **Component** | **Term** | **Estimate** | **Std Error** | **t-value** | **p-value** | |
| --- | --- | --- | --- | --- | --- | --- |
| A. parametric coefficients | (Intercept) | 6.77 | 0.08 | 90.01 | <0.001 | |
|  | SEX_M | -0.07 | 0.10 | -0.70 | 0.487 | |
| **Component** | **Term** | **edf** | **Ref. df** | **F-value** | **p-value** | |
| B. smooth terms | s(WetWT) | 1.00 | 1.00 | 45.44 | <0.001 | |
|  | s(Day of year):SEX_F | 4.50 | 8.00 | 50.65 | <0.001 | |
|  | s(Day of year):SEX_M | 4.97 | 8.00 | 39.80 | <0.001 | |
| Adjusted R-squared: 0.74, Deviance explained 74.4% | | | | | |  |
| -REML : 512.31, Scale est: 0.89, N: 362 | | | | | |  |

Table S6. Model 2b GAM results.

| **Component** | **Term** | **Estimate** | **Std Error** | **t-value** | **p-value** | |
| --- | --- | --- | --- | --- | --- | --- |
| A. parametric coefficients | (Intercept) | 7.11 | 0.10 | 74.27 | <0.001 | |
|  | SEX_M | -0.16 | 0.14 | -1.14 | 0.254 | |
| **Component** | **Term** | **edf** | **Ref. df** | **F-value** | **p-value** | |
| B. smooth terms | s(Wet weight) | 2.72 | 3.42 | 9.05 | <0.001 | |
|  | s(Day of year):SEX_F | 4.95 | 8.00 | 10.02 | <0.001 | |
|  | s(Day of year):SEX_M | 4.75 | 8.00 | 6.33 | <0.001 | |
| Adjusted R-squared: 0.48, Deviance explained 51.2% | | | | | |  |
| -REML : 383.94, Scale est: 1.01, N: 253 | | | | | |  |

Table S7. Model 2c GAM results.

| **Component** | **Term** | **Estimate** | **Std Error** | **t-value** | **p-value** | |
| --- | --- | --- | --- | --- | --- | --- |
| A. parametric coefficients | (Intercept) | 6.24 | 0.08 | 83.11 | <0.001 | |
|  | SEX_Unknown | -0.13 | 0.31 | -0.41 | 0.680 | |
|  | SEX_M | 0.09 | 0.11 | 0.81 | 0.418 | |
| **Component** | **Term** | **edf** | **Ref. df** | **F-value** | **p-value** | |
| B. smooth terms | s(Wet weight) | 1.91 | 2.48 | 21.94 | <0.001 | |
|  | s(Day of year):SEX_F | 5.27 | 8.00 | 18.83 | <0.001 | |
|  | s(Day of year):SEX_Unknown | 1.05 | 5.00 | 0.52 | 0.084 | |
|  | s(Julian_Day):SEX_M | 5.80 | 8.00 | 18.66 | <0.001 | |
| Adjusted R-squared: 0.54, Deviance explained 55.5% | | | | | |  |
| -REML : 571.66, Scale est: 1.00, N: 386 | | | | | |  |
